# Fully Compensated Ferrimagnetic Properties of (Cr,Fe)S Compound with a Pyrrhotite‐Type Structure

**DOI:** 10.1002/smll.74603

**Published:** 2026-08-03

**Authors:** Weida Yin, Masato Miyakawa, Satoshi Semboshi, Noriharu Yodoshi, Akira Masago, Yosuke Kawahito, Tetsuya Fukushima, Hisazumi Akai, Rie Y. Umetsu

**Affiliations:** ^1^ Institute for Materials Research Tohoku University Sendai Japan; ^2^ Graduate School of Engineering Tohoku University Sendai Japan; ^3^ Faculty of Materials for Energy Shimane University Matsue Japan; ^4^ Graduate School of Engineering Kyushu University Fukuoka Japan; ^5^ Japan Agency for Marine‐Earth Science and Technology Yokohama Japan; ^6^ Center for Spintronics Research Network (CSRN) Graduate School of Engineering Science Osaka University Toyonaka Japan; ^7^ Japan Agency for Marine‐Earth Science and Technology Yokosuka Japan; ^8^ Graduate School of Engineering Osaka University Suita Japan; ^9^ Research Center for Computational Design of Advanced Functional Materials National Institute of Advanced Industrial Science and Technology Tsukuba Japan; ^10^ Academia Co. Ltd. Kashiwa Japan; ^11^ Center for Science and Innovation in Spintronics Tohoku University Sendai Japan

**Keywords:** coercivity, ferrimagnet, half‐metal, magnetization compensated temperature, pyrrhotite

## Abstract

To optimize the processing conditions for the (Cr,Fe)S non‐equilibrium phase with a pyrrhotite‐type structure, the phase states and magnetic properties of the specimens obtained at various sintering temperatures were investigated. A slightly off‐stoichiometric composition of Cr_23_Fe_23_S_54_ (approximately (Cr,Fe)_7_S_8_) sintered and quenched from 1323 K indicates a single‐phase pyrrhotite‐type structure with a layered‐NiAs‐type structure in which vacancies occupy every two layers (*C*12/*c*1; the space number is 15). The compound shows fully compensated ferrimagnetic behavior at a magnetization compensated temperature of approximately 200 K. The magnetic behavior exhibits a typical N‐type ferrimagnet, as predicted by Néel. From X‐ray photoelectron spectroscopy analyses, it is found that the compound is composed of Fe^2+^ and Cr^3+^. The large magnetic coercivity of 38 kOe at 5 K is also unique and can be applied to spintronic devices. Furthermore, changing the quenching temperature enables control of the degree of order of the vacancies in the interlayer and results in tuning of the magnetization compensated temperature. First‐principles calculations show a pseudo‐gap located at the Fermi level in the up‐spin band, suggesting high spin polarization as well as the NiAs‐type structure indicated in our previous report.

## Graphical abstract

Pyrrhotite‐type crystal structure of (Cr,Fe)S compound. The fabrication conditions for obtaining a single phase of the non‐equilibrium phase were optimized. The temperature dependence of the magnetization exhibits the typical behavior of compensated ferrimagnetism. A large magnetic coercivity of the (Cr,Fe)S compound.

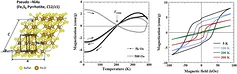

## 1 Introduction

Most half‐metals reported thus far are ferromagnets. However, half‐metals with alternative magnetic characteristics can lead to the development of new spintronic devices. One such case, proposed by van Leuken and de Groot in 1995 [1], is half‐metallic fully compensated ferrimagnets (HM‐FCFiMs). Several types of materials have been predicted to be HM‐FCFiMs based on the theoretical calculations of perovskite oxides (La_2_ *M*′*M*″O_6_: *M*′, *M*″ = transition metal) [2], double perovskites (La*A*VRuO_6_: *A* = Ca, Sr, and Ba) [3], and Cr‐based full‐ and half‐Heusler alloys (Cr_2_Mn*Z* (*Z* = P, As, Sb, and Bi) [4], CrMn*Z* (*Z* = P, As, and Sb) [5]). Akai and Ogura predicted the possible existence of HM‐FCFiMs in diluted magnetic semiconductors [6] and pnictide or chalcogenide compounds with NiAs‐type crystal structures [7].

Recently, several materials showing potential as HM‐FCFiMs have been experimentally reported, such as Mn‐based bulk Heusler alloys of Mn‐V‐Fe‐Al (Mn_1.5_V_0.5_FeAl [8], Mn_1.2_Fe_1.18_V_0.62_Al [9]), Mn_2_V_0.5_Co_0.5_Al melt‐spun ribbons [10], and Mn_2_Ru*_x_* Ga thin film [11]. *L*2_1_‐type crystal cells have been determined to exhibit N‐ or P‐type ferrimagnets, as predicted by Néel [12]. A new material for HM‐FCFiMs without Heusler alloys was reported for the first time by the authors of the previous paper [13]. The material was a (Cr,Fe)S compound with a NiAs‐type structure, and the composition was designed based on the concept that the total number of *d*‐electrons per magnetic ion must be equal to five [14].

Many types of compounds have been reported considering the equiatomic compositions of Cr‐S, Cr‐Se Fe‐S, and Fe‐Se systems, and superconducting FeSe is a well‐known material [15]. Although the crystal structure of FeSe is layered, these materials exhibit a hexagonal NiAs‐type structure, which includes vacancies in the equilibrium state at an off‐stoichiometric composition and has a long‐range stacking structure [16, 17]. The phases of Cr_2_S_3_, Cr_3_S_4_, Cr_5_S_6_, and Cr_7_S_8_ are in the Cr‐S system, while those of Fe_7_S_8_, Fe_9_S_10_, Fe_10_S_11_, and Fe_11_S_12_ are in the Fe‐S system, considering the binary phase diagram [18, 19]. A phase of the NiAs‐type structure exists at high temperature ranges in both phase diagrams of Cr‐S and Fe‐S systems around the equiatomic composition, and the phase can exist as a solid solution system from the ternary phase diagram of Cr‐Fe‐S [20]. Over the last 50 years, FeCr_2_S_4_ chalcogenide spinel compounds, where a part of the Fe is substituted by another transition element, have attracted attention for their large magnetoresistance [21], dynamic and static Jahn–Teller effects [22, 23, 24, 25, 26], topological anomalous Hall effects [27], multiferroicity [28, 29], and magnetostructural transformations [30, 31, 32]. This compound is stable at low temperatures. However, in our previous study, a new non‐equilibrium phase with a NiAs‐type crystal structure was identified based on the concept proposed by Akai et al., which is related to the number of *d*‐electrons in this system.

In our previous study, slightly off‐stoichiometric (Cr,Fe)S with a NiAs‐type crystal structure was successfully obtained via powder solid sintering and quenching at high temperatures [13]. The magnetic data showed that total magnetization compensated at approximately 200 K, and the thermomagnetization curve behaved as an N‐type ferrimagnet predicted by Néel, although it shifted slightly to a P‐type ferrimagnet under the application of a larger magnetic field [12]. Among the predicted candidates, (Cr,Fe)S is attractive because it is composed of common elements with high Clarke numbers. Although the composition was optimized over a wide composition range [13], sintering and quenching were not performed. Because a small amount of the secondary phase was included in our previous report, the process used to obtain these materials must be further investigated.

In this study, the quenching temperature dependences of the phase state, crystal structure, and magnetic properties of Cr_23_Fe_23_S_54_ were investigated to optimize the processing conditions of the compound. It is found that the crystal structure of the obtained single phase is a pseudo‐NiAs‐type (pyrrhotite‐type) structure with stacking by careful analysis in the lower angle region via X‐ray diffraction experiments. X‐ray photoelectron spectroscopy analyses also reveal that the materials consist of Fe^+2^ and Cr^+3^.

## 2 Experimental Procedures

**FIGURE 1:**
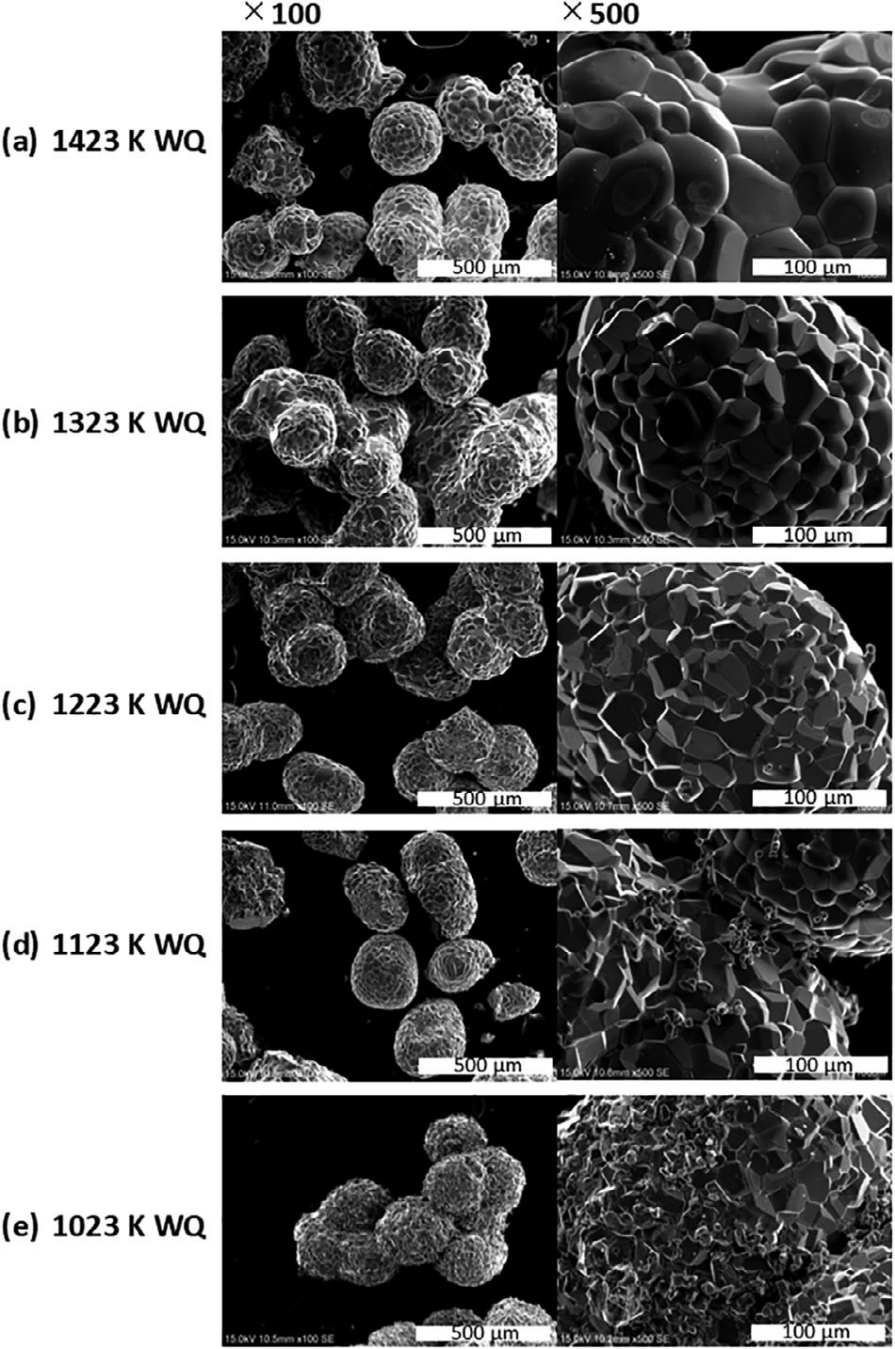
Microstructures observed via SEM for the specimens obtained by sintering and quenching at various temperatures ranging from (a) 1423 K to (e) 1023 K.

Cr_23_Fe_23_S_54_ was synthesized using powder metallurgy. Raw elemental powders of pure Cr, Fe, and S were mixed and compressed via cold uniaxial pressing of 30 MP at room temperature. The obtained cylindrical compact was encapsulated in a quartz tube under an Ar atmosphere, and the furnace temperature was gradually increased to the expected temperature. After reaching the sintering temperature, the compact was sintered for 1 d and then quenched. The sintering temperatures used were 1023 K, 1123 K, 1223 K, 1323 K, and 1423 K. The microstructure and composition were examined using scanning electron microscopy (SEM) and inductively coupled plasma (ICP) analysis, respectively. The crystal structure was evaluated using X‐ray diffraction experiments with Co‐*K*α radiation. Magnetic measurements were performed using a superconducting quantum interference (SQUID) magnetometer and a vibrating sample magnetometer based on a physical property measurement system (Quantum Design, Ltd.). The size of each granule is several hundred microns, as shown in Figure 1. The microstructure images do not show any particular orientation of individual crystal grains, so it can be considered a perfectly polycrystalline material. The measured magnetization value is on the order of 10^−^^4^ – 10^−^^2^ emu, and the demagnetizing field is negligibly small; therefore, demagnetizing field correction is unnecessary.

The Cr_23_Fe_23_S_54_ powders, heat‐treated at 1323 and 1423 K, were pestled and pressed into pellets under a pressure of 30 MPa. The pellet‐shaped samples were subsequently embedded in resin and polished. X‐ray photoelectron spectroscopy (XPS) measurements were carried out using a Shimadzu/KRATOS AXIS‐Ultra DLD system to evaluate the valence of the constituent elements of Fe and Cr. The energy calibration was performed by setting the C─C and C─H bonds to 284.8 eV.

## 3 Theoretical Calculations

Theoretical calculations were performed using the first‐principles calculation package AkaiKKR, in which the all‐electron Korringa–Kohn–Rostoker Green's function method was implemented [33]. Calculations were conducted using the atomic sphere approximation, in which overlapping spherically symmetric potentials were employed. In this study, we used the local density approximation with the generalized gradient approximation proposed by Perdew, Burke, and Ernzerhof for the exchange‐correlation functional [34]. Furthermore, we employed both non‐relativistic and scalar relativistic approximations. However, no significant differences were observed among the results.

The model structure was assumed to be of the pyrrhotite‐type, characterized by the space group *C1*2/*c1*. This prototype structure contains 14 Fe atoms and 16 S atoms per unit cell. The Fe sites are partially occupied by Cr atoms. This configurational disorder was treated using coherent potential approximation [35, 36]. The atomic positions were set consistent with the current experimental structural analysis. Eight spin configurations were considered: (i) ferromagnetic alignment of the magnetic moments for all Fe atoms, and (ii) seven different collinear antiferromagnetic configurations for the Fe atoms. These configurations were uniquely determined by the symmetry of the *C*12/*c*1 structure. The Fe and Cr spins located on the same type of site were forced to couple antiparallel to each other in all configurations.

## 4 Experimental Results

Figure 1 shows SEM images of the microstructures of the specimens. The sintering and water quenching (WQ) temperatures were varied from 1023 K to 1423 K (the specimens are denoted as 1023 K WQ). The size of one granule was several hundred microns, and it is composed of particles approximately 20–40 µm in size. Smaller particles of less than 10 µm can also be seen in the specimens obtained from lower quenching temperatures, but these may be attributed to the secondary phase. The number of small particles decreased with increasing quenching temperature, and no such small particles were observed in the specimens quenched at 1323 K and 1423 K. It is clear that a single phase can be obtained by quenching at higher temperatures. By comparing the microstructures of the specimens obtained at 1323 K and 1423 K, the outside appearances were not very different, and it seems that the grain size was slightly larger at 1423 K than at 1323 K. The compositions evaluated using ICP are listed in Table 1. The results indicate that all obtained specimens exhibit a nominal composition, and the error bars result from the difficulty in evaluating S using the ICP method.

**TABLE 1:** Compositions of Cr_23_Fe_23_S_54_ compounds sintered and quenched from various temperatures evaluated using the ICP method.

Sintering and quenching temperature [K]	Cr [at.%]	Fe [at.%]	S [at.%]
1423	22.6	22.7	54.7
1323	24.5	21.8	53.7
1223	23.9	22.4	53.7
1123	23.4	22.8	53.8
1023	24.7	21.0	54.3

**FIGURE 2:**
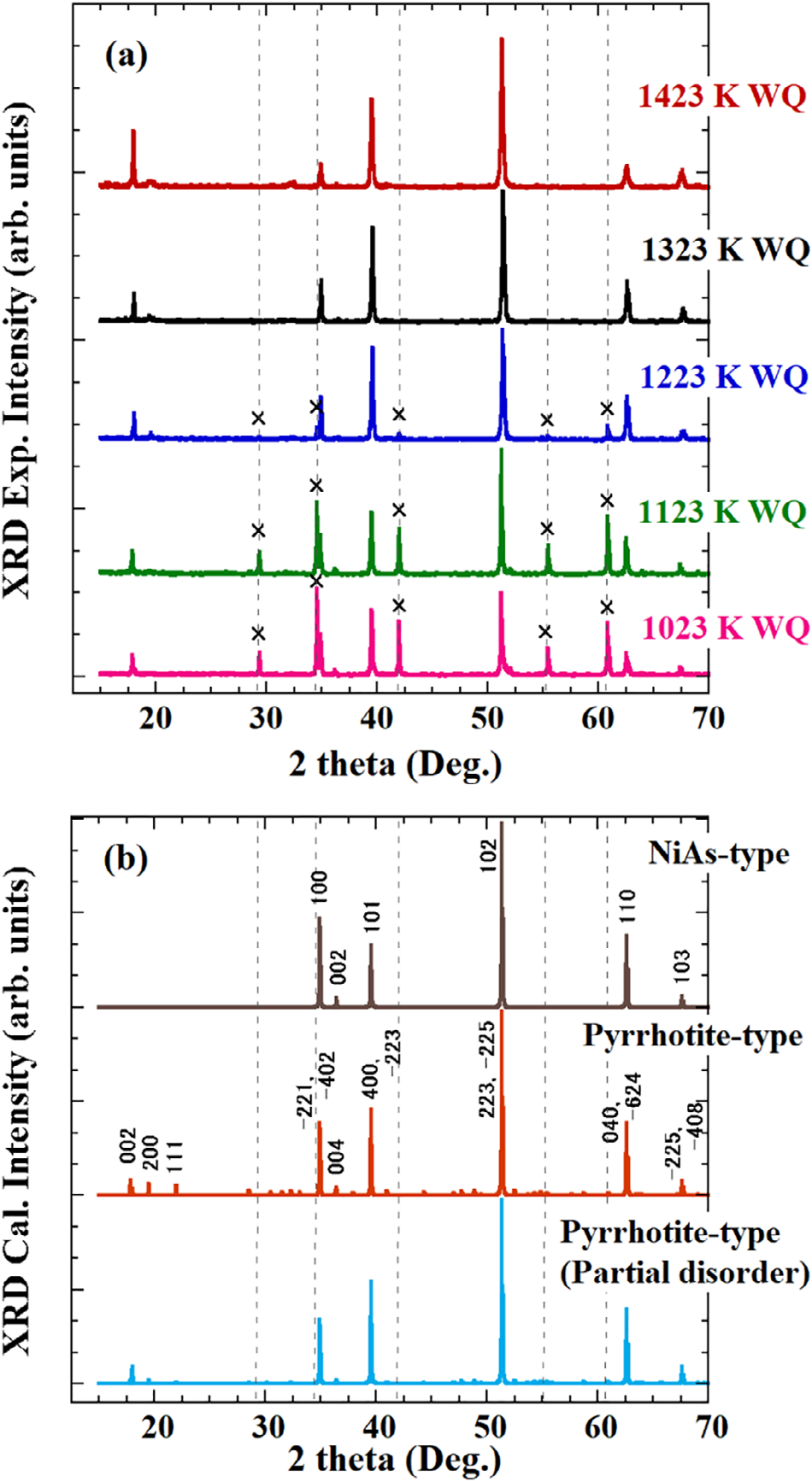
(a) XRD patterns for Cr_23_Fe_23_S_54_ compounds sintered and quenched at various temperatures measured using a Co‐*K*α radiation source at room temperature. Peaks indicated with crosses (x) correspond to the secondary phase. (b) Simulated XRD patterns for the (Cr,Fe)S NiAs‐type structure and (Cr,Fe)_7_S_8_ with a pyrrhotite‐type structure. The middle pattern colored by orange was obtained under the assumption that vacancies are ordered at a certain position (4e site. The lowest pattern colored by light blue was obtained assuming that the vacancies were partially disordered within the layer.

**FIGURE 3:**
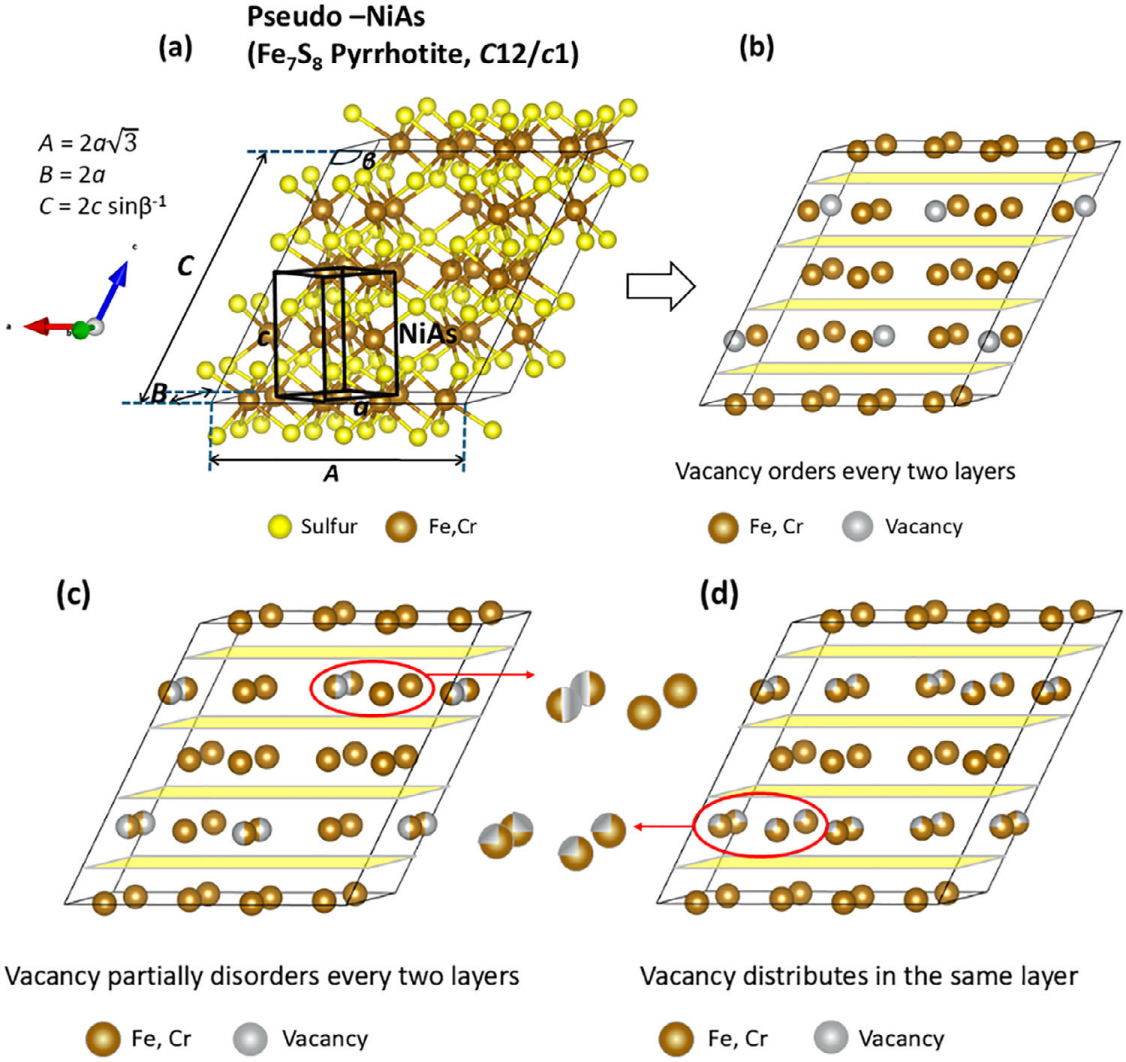
Crystal structure of the prototype pyrrhotite structure (Fe_7_S_8_) containing along‐range stacking of the NiAs structure, as indicated by the solid line. Here, (a) and (b) show the same structure. Here, the layers constructed of S atoms are indicated as a yellow layer simply; in addition, the vacancies are shown in gray in (b). The vacancies are assumed to be disordered inside each layer in (c) and (d). They partially disorder in (c), but they perfectly disorder inside the layer in (d).

Figure 2a shows XRD patterns measured at room temperature using Co‐*K*α radiation for Cr_23_Fe_23_S_54_ compounds, which were sintered and quenched at various temperatures from 1023 K to 1423 K. Figure 2b shows the simulated XRD patterns of the NiAs‐type hexagonal and pyrrhotite‐type structures. The pyrrhotite‐type structure (*C*12/*c*1; space number: 15; prototype: Fe_7_S_8_) was a layered NiAs‐type structure (*P*63/*mmc*; space number: 194). Most importantly, vacancies exist instead of 1/8 Fe atoms. Therefore, the two theta positions at which the main peaks were observed were the same. These systems differed in the existence of peaks around 2*θ* = 20°, and small peaks were distributed throughout the spectra. Comparing the experimentally obtained XRD patterns and the simulated patterns shown in Figure 2b, the extra peaks denoted by crosses (×) for the specimens obtained at quenching temperatures below 1223 K did not correspond to either structure. The peaks of the secondary phase can be attributed to the FeCr_2_S_4_ chalcogenide spinel compound because this phase is stable at low temperatures, as shown in the ternary phase diagram of this system [20]. Here, the lattice parameters in the simulated XRD patterns are assumed to be *a* = 0.3450 and *c* = 0.5723 nm in the NiAs‐type structure, and *A* = 2*a* $\sqrt{3}$ = 1.1942 nm, *B* = 2*a* = 0.6885 nm, *C* = 2*c* (sin*β*)^−1^ = 1.2905, and *β* = 117.5 in pyrrhotite. The crystal structures of NiAs‐ and pyrrhotite‐type structures are shown in Figure 3a. The pyrrhotite‐type structure is layered two times in the directions of the *A* and *C* axes. Figure 3b shows a simplified pyrrhotite‐type structure, in which the layers consisting of S are shown in the yellow layer. The positions of the vacancies are readily observed at Fe/Cr layers. Here, it is confirmed that the vacancies are ordered every two layers. In the experimentally obtained XRD patterns, it was found that the peak positions around 2*θ* = 20° coincide with those of the simulated XRD pattern; however, the intensity differed. To investigate the intensities of the 002, 200, and 111 diffraction peaks, partial disordering of the vacancies was introduced in the XRD simulation. The best fit was obtained as indicated by the lowest patterns colored by light blue shown in Figure 2b. Here, the vacancy is assumed to be disordered between the ordinate position and the nearest neighbor in the same layer; the structure is shown in Figure 3c. The detailed fitting parameters are provided in the Supporting Information. Here, the *S* values, meaning the goodness of fit, were 1.43 and 1.30 for the two specimens, respectively. Furthermore, the intensity of the 002 diffraction peak increased in the specimen obtained at 1423 K WQ. Here, further disordering of the vacancy was introduced, as shown in Figure 3d, and the vacancies were distributed in the same layer. Interestingly, changing the quenching temperature resulted in a change in the degree of vacancy ordering. It should be noted that in all simulations, it was assumed that the Cr and Fe atoms were always disordered. This assumption was based on the results obtained from the Mössbauer experiments reported by Sokolovich and Bayukov [37]. They investigated the Mössbauer spectra of the compounds in the CrS–FeS system over a wide range of compositions. The specimens were obtained by rapid cooling, and spectral analysis showed that the system was a substitutional solid solution with a random distribution of Cr and Fe atoms. Since Mössbauer is very sensitive to the local structure of Fe, we refer to the reported results here.

**FIGURE 4:**
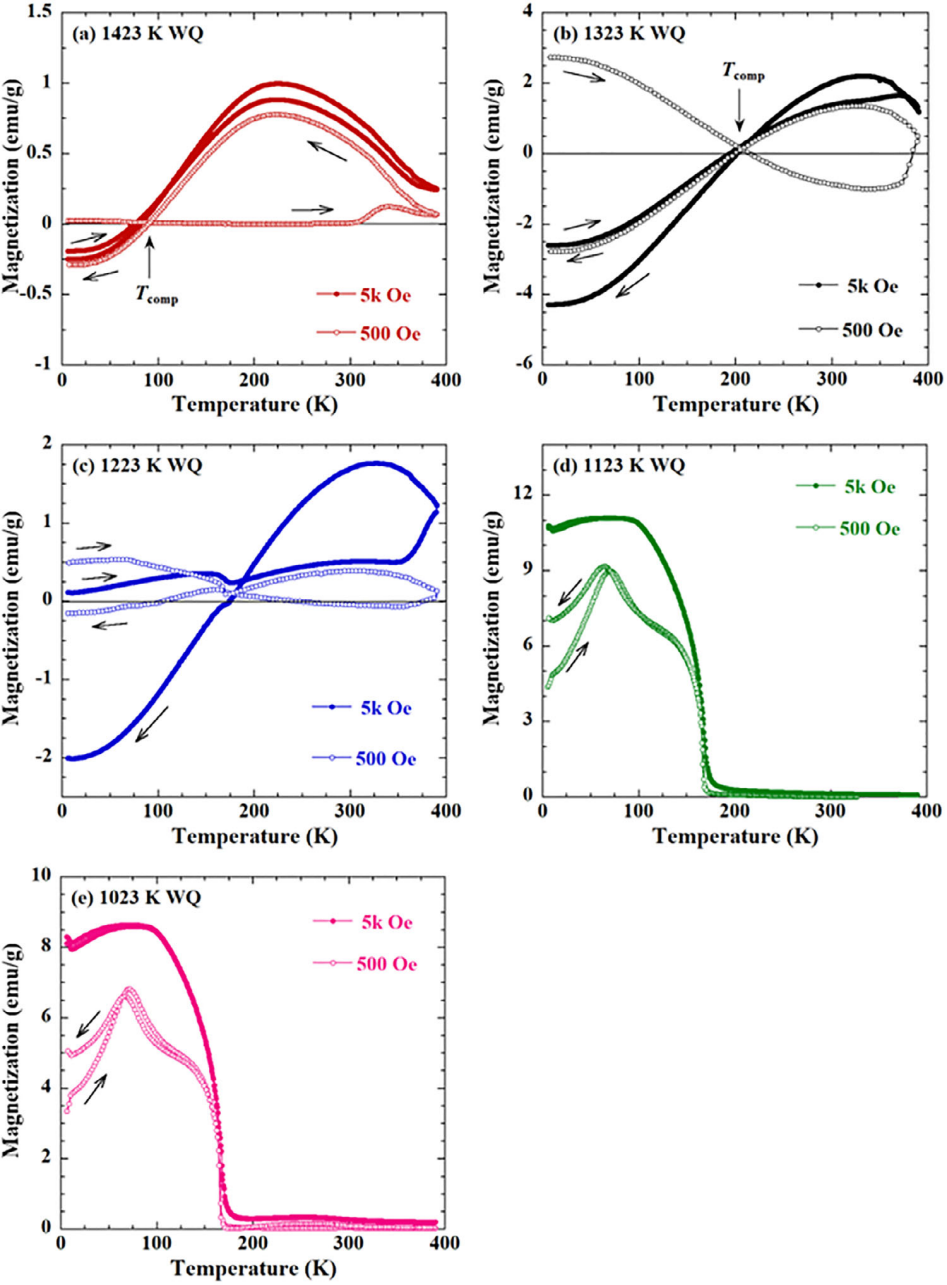
Thermomagnetization curves measured under magnetic fields of 500 and 5 kOe. Figures (a–e) correspond to the specimens obtained by quenching at 1423 K, 1323 K, 1223 K, 1123 K, and 1023 K, respectively.

Figure 4 shows the thermomagnetization (*M*‐*T*) curves measured under magnetic fields of 500 and 5 kOe for each specimen. The *M‐* *T* behavior roughly differed between the specimens obtained at lower‐ and higher‐temperature quenching and is consistent with the XRD results and microstructural observations. For the 1423 K WQ specimen, the *M*–*T* curves show a completely different behavior between the heating and cooling processes under a 500 Oe magnetic field, whereas those in the heating and cooling processes at 5 kOe were overlaid upon one another. The cross point observed around 90 K contributes to the magnetization‐compensated temperature; here, the magnetization of the two sublattices cancel each other. For the specimen obtained at 1323 K WQ, the typical N‐type behavior of the *M*–*T* curve, as predicted by Néel, was observed. The Curie temperature is expected to be slightly higher than 400 K. The N‐type behavior and another ferri/ferromagnetic component were mixed in the 1223 K WQ specimen. It is clear that the additional magnetic component originated from the magnetic behavior of the FeCr_2_S_4_ spinel compound. The *M‐* *T* behavior of the 1123 K and 1023 K WQ specimens shows a cusp around 70 K, and the Curie temperature is approximately 170 K. The field cooling effect is observed below 70 K, and these behaviors coincide with the reported magnetic properties of the FeCr_2_S_4_ spinel compound [38, 39].

**FIGURE 5:**
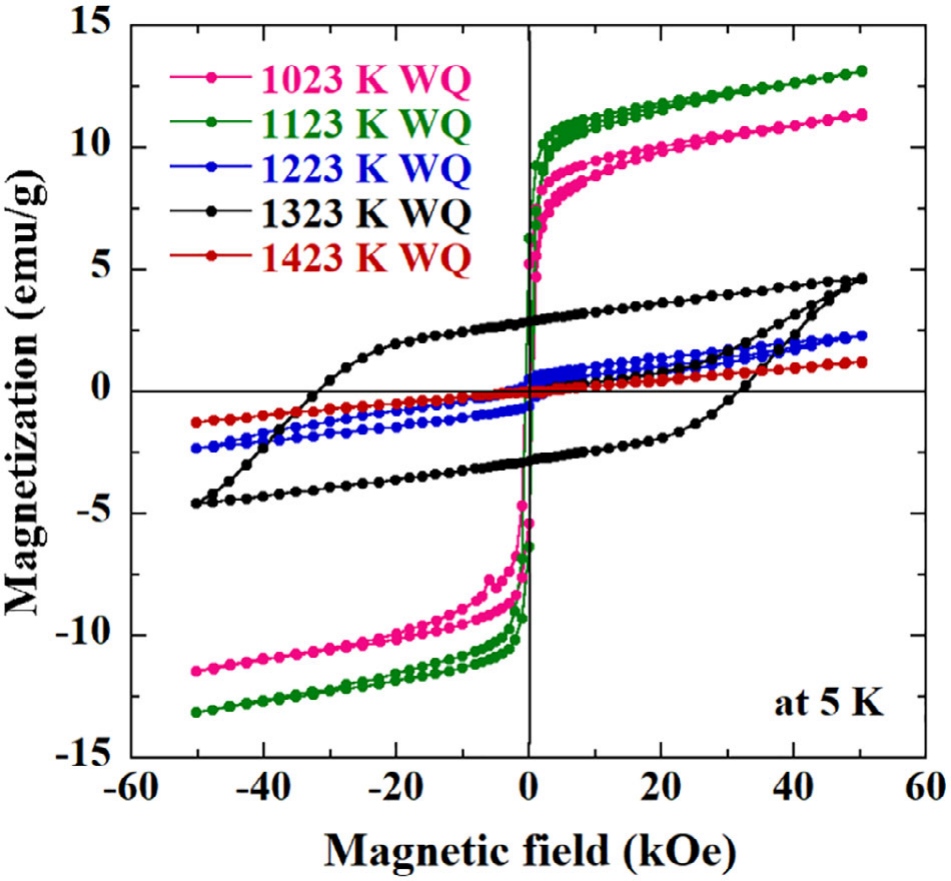
Magnetization curves measured at 5 K up to a magnetic field of 50 kOe for each specimen obtained at different quenching temperatures.

The magnetization (*M*–*H*) curves of the series of specimens measured at 5 K are shown in Figure 5. The specimens obtained at 1023 K and 1123 K WQ show ferrimagnetic‐like behavior, and the magnetization reached 12–14 emu/g at 50 kOe. It has been reported that the magnetic moment of Cr^3+^ and Fe^2+^ is antiferromagnetically coupled, and the values of the magnetic moments are 2.9 μ_B_ and 4.2 μ_B_ at 4.2 K, respectively, providing a total magnetic moment of 1.6 μ_B_ per formula unit [40, 41]. Although the physical properties of the spinel have attracted much research attention [21, 22, 23, 24, 25, 26, 27, 28, 29, 30, 31, 32], spinel is the secondary phase in the present analysis, and it is therefore not discussed in depth. The magnetic component of the spinel decreases with increasing quenching temperature. The specimens obtained at 1323 K and 1423 K WQ are single‐phase, and the *M*–*H* curve of the 1423 K WQ specimen showed a linear behavior, while the 1323 K WQ sample demonstrated an extremely large coercivity *H*_c_. The *M*–*H* curve for 1323 K WQ does not appear to saturate even at a magnetic field of 50 kOe; therefore, the magnetization was measured up to 90 kOe for the sample.

**FIGURE 6:**
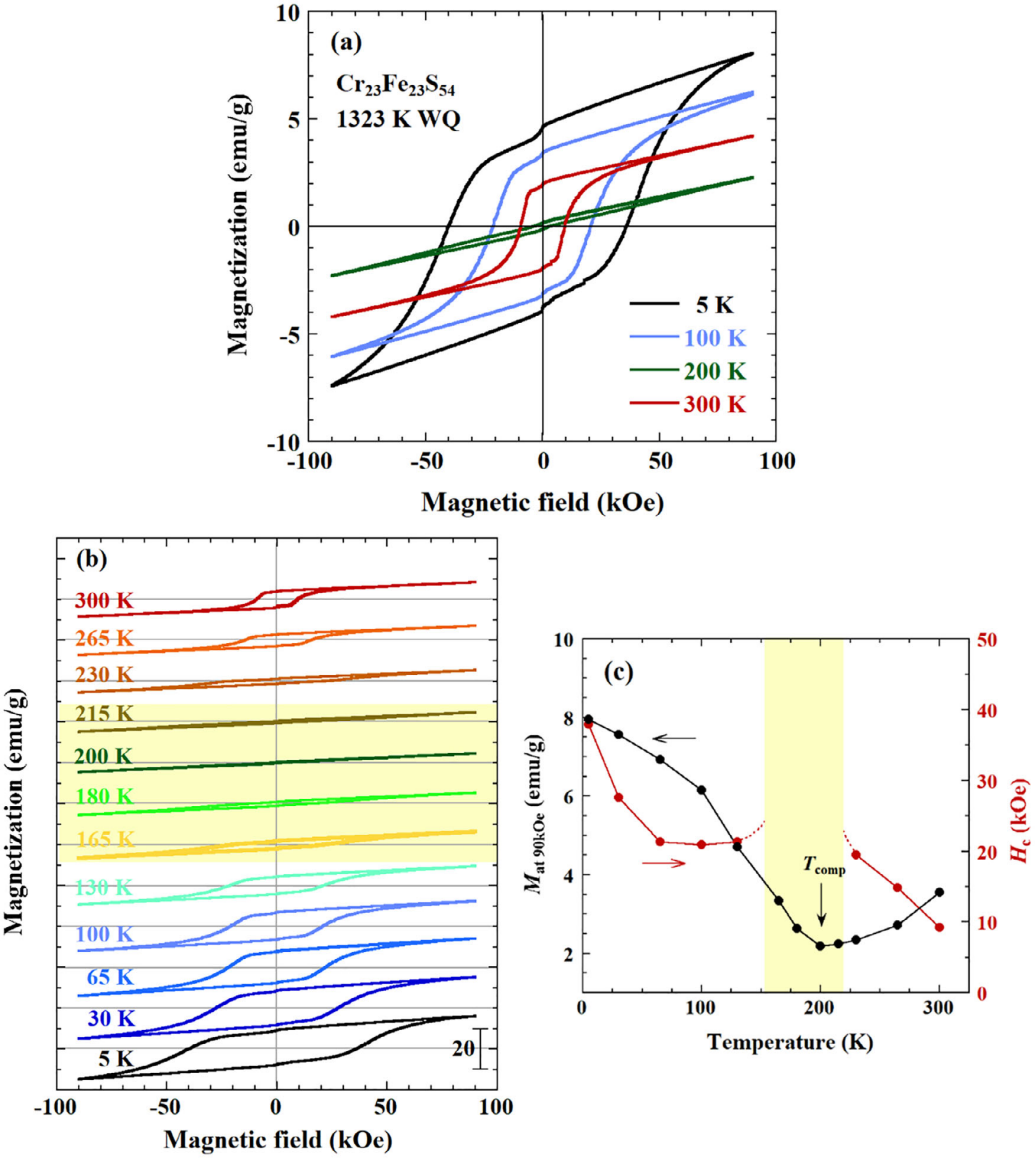
(a) Magnetization (*M‐* *H*) curves measured at 5 K, 100 K, 200 K, and 300 K for the specimen of sintered and quenched at 1323 K. (b) *M*–*H* curves obtained at temperatures ranging from 5 K to 300 K. (c) Temperature dependences of the magnetization obtained at 90 kOe (*M*_90 kOe_), and the coercivity (*H*_c_). (b) and (c) are also obtained for the specimen of 1323 WQ.

Figure 6a shows the *M*–*H* curves of the 1323 K WQ specimen measured at 5 K, 100 K, 200 K, and 300 K, and Figure 6b that for the entire temperature range from 5 K to 300 K. The hysteresis loop measured at 5 K becomes closed at approximately 80 kOe, and *H*_c_ reaches 38 kOe. *H*_c_ decreases with increasing temperature, is near zero in the vicinity of magnetization compensated temperature *T*_comp_, and increases above this temperature. A similar tendency was also observed in our previous report [13], however, it is thought that the small *H*_c_ is due to the minor loop behavior. It is known that *H*_c_ diverges at *T*_comp_ in ferrimagnetic systems, such as rare earth compounds and oxides [42, 43, 44, 45, 46, 47]. To determine *H*_c_ near *T*_comp_, a significantly higher magnetic field is required. Figure 6c indicates the temperature dependences of *H*_c_ and magnetization at 90 kOe (*M*_90 kOe_). Here, *H*_c_ is not encountered around *T*_comp_. In the *M–H* curve at 5 K for the 1423 K WQ specimen (Figure 5), a linear tendency is observed. This could also be associated with the minor loop in this curve because *T*_comp_ is approximately 90 K, which is lower than that for the 1323 K WQ specimen. Although the divergence of *H*_c_ in the vicinity of *T*_comp_ was not observed in the present polycrystalline specimen, the symptom can be observed in the single crystal of (Cr,Fe)_7_S_8_ obtained by the chemical vapor transport method, which was successfully obtained by our group. The physical properties of these single crystals will be investigated in a future study. The magnetization obtained at 5 K and 90 kOe was approximately 8 emu/g, which was converted to approximately 0.9 *μ*_B_/f.u. in the pyrrhotite‐type structure.

**FIGURE 7:**
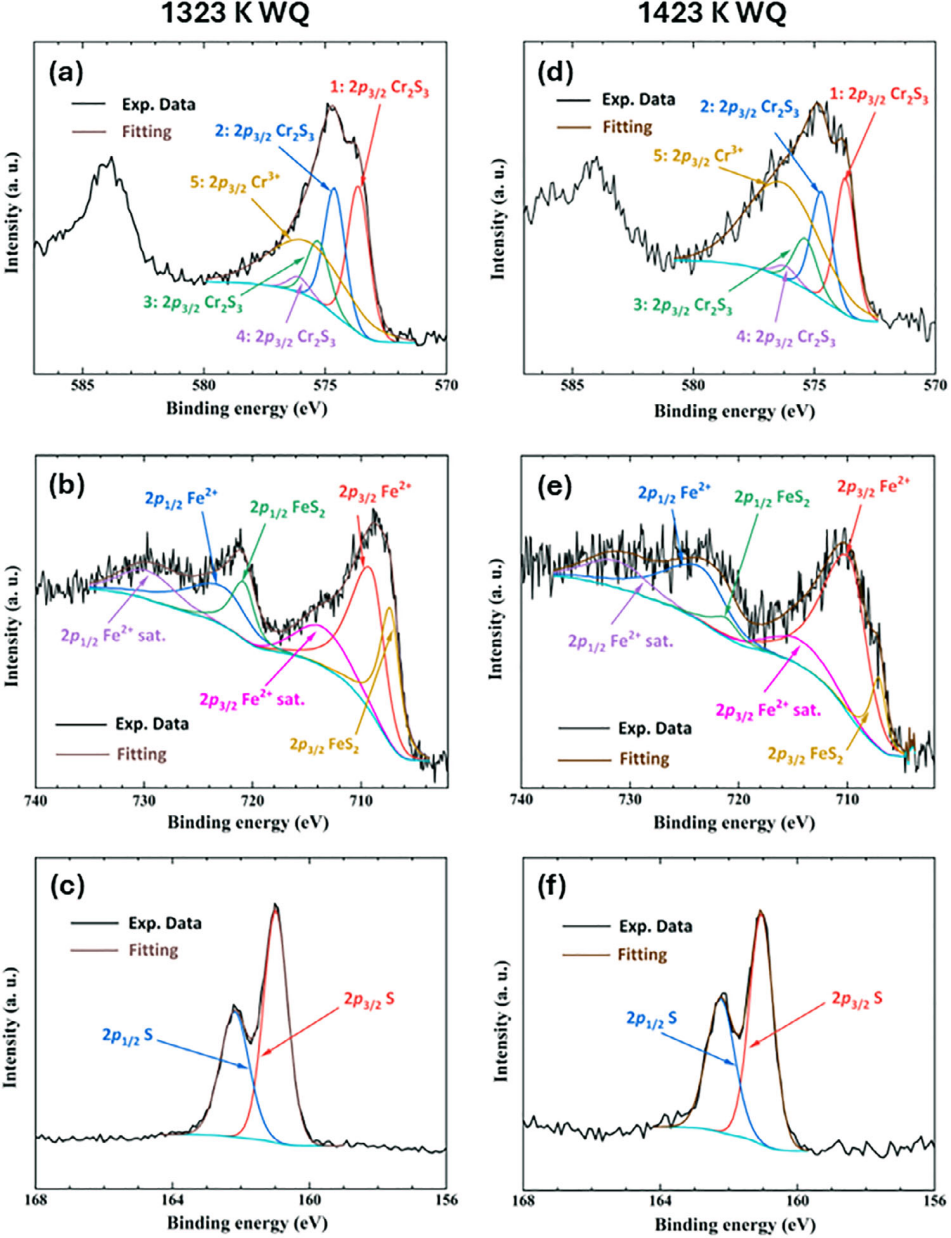
XPS analysis of Cr, Fe, and S in Cr_23_Fe_23_S_54_ sintered at (a)–(c) 1323 K and (d)–(f) 1423 K.

Figure 7 shows the XPS results of Cr, Fe, and S for Cr_23_Fe_23_S_54_, which was sintered and quenched from 1323 K (Figure 7a–c) and 1423 K (Figure 7d–f). In Figure 7a,d, broad peaks associated with Cr 2*p*_3/2_ are observed around the binding energy of 575 eV. Here, this system was analyzed considering the reference spectra provided in previous studies [48, 49]. The broad peak results from the sum of five peaks, and the peak positions are 573.64 eV, 574.62 eV, 575.29 eV, 576.10 eV, and 575.7 eV in Figure 7a. Here, the first four peaks correlate to those of Cr_2_S_3_ [48], and the last may result from Cr_2_O_3_. Therefore, the obtained spectra result from Cr^3+^. The analysis presented in Figure 7d was conducted in the same manner, wherein the results are similar to those obtained for the 1323 K WQ specimen. The numerical results of these analyses are listed in Table 2.

**TABLE 2:** XPS spectra analysis results. The upper and lower tables corresponded to the specimens obtained at 1323 K WQ and 1423 K WQ, respectively.

	No.	Name	Position	FWHM	Sub total [%]	Total [%]
1323 K		Fe 2*p*_3/2_ 2+	709.04	2.83	18.6	26.2
			Fe 2*p*_1/2_ 2+	722.64	5.2	
			Fe 2*p*_3/2_ 2+ SAT	713.56	5.65	
			Fe 2*p*_1/2_ 2+ SAT	729.68	6	
			Fe 2*p*_3/2_ FeS_2_	707.29	1.98	7.6
			Fe 2*p*_1/2_ FeS_2_	720.81	2.33	
	1	Cr 2*p*_3/2_ Cr_2_S_3_	573.64	1.05	13.7	21.7
	2	Cr 2*p*_3/2_ Cr_2_S_3_	574.62	1		
	3	Cr 2*p*_3/2_ Cr_2_S_3_	575.29	1.09		
	4	Cr 2*p*_3/2_ Cr_2_S_3_	576.10	1		
	5	Cr 2*p*_3/2_ 3+	575.70	3.85	8.0	
		S 2*p*_3/2_ Sulfide	160.98	0.84	52.1	52.1
		S 2*p*_1/2_ Sulfide	162.18	0.99		
	No.	Name	Position	FWHM	Sub total [%]	Total [%]
1423 K		Fe 2*p*_3/2_ 2+	709.78	3.62	22.9	25.9
		Fe 2*p*_1/2_ 2+	723.38	5.24		
		Fe 2*p*_3/2_ 2+ SAT	714.40	6.56		
		Fe 2*p*_1/2_ 2+ SAT	731.26	6.58		
		Fe 2*p*_3/2_ FeS_2_	707.10	1.53	3.0	
		Fe 2*p*_1/2_ FeS_2_	721.18	2.76		
	1	Cr 2*p*_3/2_ Cr_2_S_3_	573.72	0.97	10.8	22.5
	2	Cr 2*p*_3/2_ Cr_2_S_3_	574.70	1.01		
	3	Cr 2*p*_3/2_ Cr_2_S_3_	575.37	1.13		
	4	Cr 2*p*_3/2_ Cr_2_S_3_	576.18	1.03		
	5	Cr 2*p* 3+	576.30	3.56	11.7	
		S 2*p*_3/2_ Sulfide	161.05	0.82	51.6	51.6
		S 2*p*_1/2_ Sulfide	162.23	0.99		

Figure 7b,e shows the XPS spectra of Fe2*p* at 1323 and 1423 K WQ, respectively. These peaks are composed of Fe2*p*_3/2_ and Fe2*p*_1/2_ and their satellites. The main peak at approximately 709 eV can be assessed via the spectra of Fe2*p*_3/2_, in which FeS_2_ and FeO appear at 707 eV and 709.04 eV, respectively [50]. This means that the covalent nature of Fe is Fe^2+^, and the species are the same as in the spectrum of the 1423 K WQ specimen. The roles of Cr and Fe are defined via their valence. Figure 7c,f shows the XPS spectra for S2*p*, exhibiting the species S2*p*_3/2_ and S2*p*_1/2_. A comparison between the two samples obtained at different quenching temperatures revealed that the intensities of the Cr_2_S_3_ and FeS_2_ peaks were reduced in the 1423 K WQ sample. This suggests that the Fe/Cr environment differed between the two specimens. XRD analyses showed that the vacancies in each of the two layers tended to be disordered in the interlayer at 1423 K WQ, which resulted in a higher quenching temperature. A small difference in the environment causes a balance in the strength of the sublattice magnetization, which leads to different *T*_comp_ values.

## 5 Calculation Results

**FIGURE 8:**
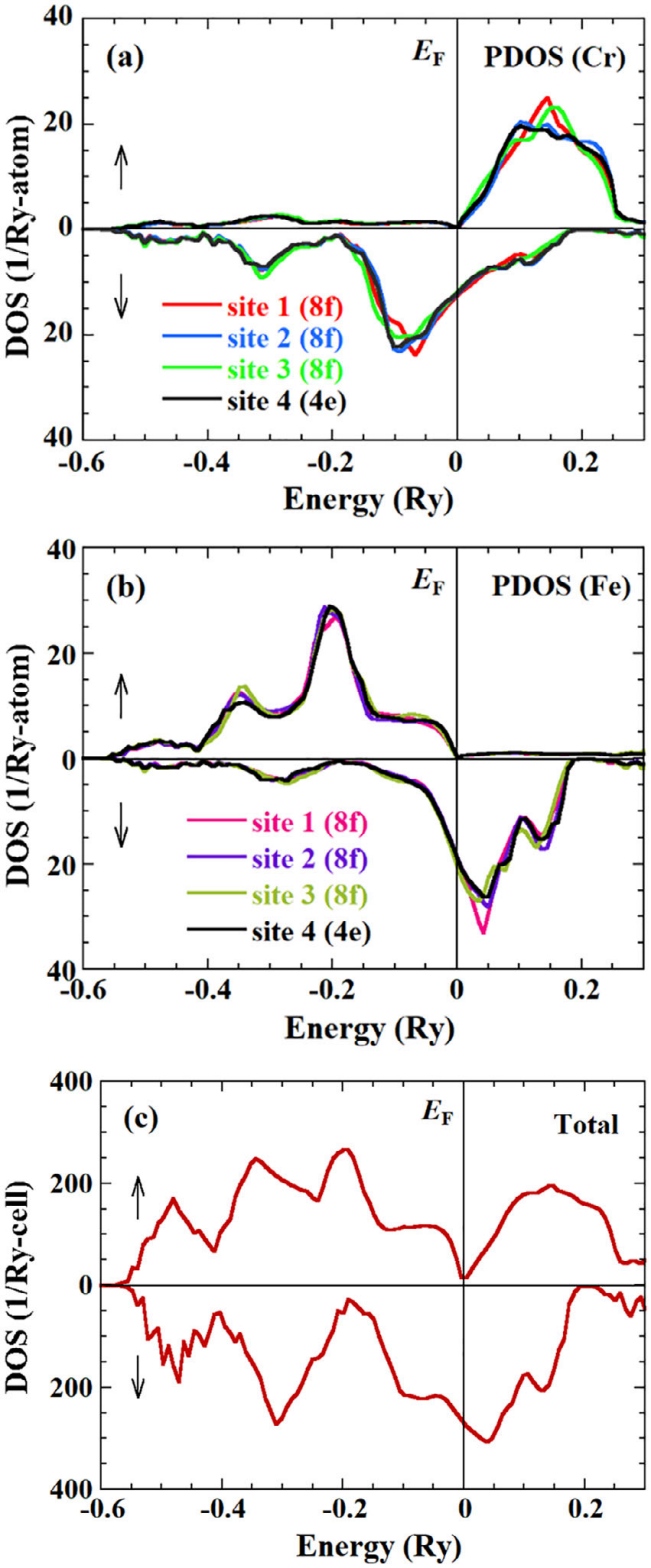
Partial density of states (DOS) of Cr (a) and Fe (b) and the total DOS (c) of (Cr,Fe)_7_S_8_. Here, Cr and Fe are assumed to be randomly distributed on the Fe sites in Fe_7_S_8_ pyrrhotite structure. The atomic positions of each element are set to be the same as those in the prototype Fe_7_S_8_.

Our calculations showed that the parallel‐spin configuration was the most stable for all Fe atoms. Because Fe and Cr align antiparallelly at the same type of site and each possesses a nonzero magnetic moment, a ferrimagnetic spin configuration was realized. The energy differences among the eight spin configurations were relatively small, on the order of several hundred meV per unit cell. Figure 8a–c shows the partial density of states (DOS) of Cr and Fe and the total DOS of (Cr,Fe)_7_S_8_, respectively, for the most stable state. The positive side of the vertical axis shows the majority‐spin states, whereas the negative side represents the minority‐spin states. In addition, the horizontal axis indicates the energy relative to the Fermi level. Here, Cr and Fe were assumed to be randomly distributed at each of the three 8*f* sites and one 4*e* site. The atomic positions of each element were set to be the same as those of the prototype Fe_7_S_8_ pyrrhotite [51], and the lattice parameters were used to obtain the values from the present XRD refinement (*A* = 1.1942, *B* = 0.6885, and *C* = 1.2905 nm). The values of the magnetic moments of Cr and Fe at each of the four aforementioned sites were −2.91, −2.87, −2.99, and −2.86 *μ*_B,_ and 3.25 *μ*_B_, 3.31 *μ*_B_, 3.19 *μ*_B_, and 3.25 *μ*_B_, and these values are insensitive to the positions. The S elements also exhibited small values, and the total magnetic moment was calculated as 3.54 *μ*_B_/f.u. The calculated magnetic moments for each element are listed in Table 3.

**TABLE 3:** Magnetic moments obtained via first‐principles calculations. The positions of atoms were obtained from the crystallographic information files for Fe_7_S_8_ pyrrhotite (*C*12/*c*1) [51]. Here, the lattice parameters obtained from the present XRD analysis were used, and Fe and Cr atoms were assumed to be distributed randomly at each Fe site.

	Fe [*μ*_B_]	Cr [*μ*_B_]		S [*μ*_B_]
Site Fe1 (8*f*)	3.25	−2.91	Site S1 (8*f*)	0.10
Site Fe 2 (8*f*)	3.31	−2.87	Site S2 (8*f*)	0.09
Site Fe 3 (8*f*)	3.19	−2.99	Site S3 (8*f*)	0.09
Site Fe 4 (4*e*)	3.25	−2.86	Site S4 (8*f*)	0.12

The total DOS shown in Figure 8c demonstrates that a gap appeared exactly at the Fermi level in the majority‐spin states, where the DOS became zero, indicating a half‐metallic state. Therefore, these results suggest that (Cr,Fe)_7_S_8_ is a potential HM‐FCFiM system. The spin polarization of (Cr_0.5_Fe_0.5_)S with a NiAs‐type structure was found to be 99.7% in our previous study [13]. A high spin polarization is maintained in the pyrrhotite structure, which is a stacked NiAs‐type structure. Both Fe and Cr have high‐spin configurations, and the crystal field effect of these components is weaker than that of pyrite, a common Fe‐S compound. The well‐defined *e*_g_‐*t*_2g_ crystal field splitting characteristics of pyrite and similar compounds are absent in this system because of its lower symmetry and more anisotropic ligand environment. This half‐metallic state arises because the majority‐spin Fe‐*d* orbitals are fully occupied, whereas the majority‐spin *d* orbitals of Cr are largely unoccupied. This half‐metallic characteristic results from the ferromagnetic alignment of the local states. If the Fe local moments were aligned antiferromagnetically, the system would form an antiferromagnetic superposition of half‐metallic states, and the spin‐dependent gap in the total DOS would vanish. Because the energy difference between the parallel and antiparallel Fe spin configurations calculated in this study is very small, the stable spin configuration may be altered via temperature, atomic position fluctuations, or the presence of vacancies. Additionally, because the spins of Fe and Cr are antiparallel, the total magnetic moment is relatively small, and some of these derivatives may include altermagnets with net magnetic moments of zero. The calculations in this paper used lattice constants and atomic positions obtained from the experiments at room temperature. Naturally, these change with temperature, and this will sensitively affect the magnetic properties. Furthermore, the present calculations were performed under the prototype of the pyrrhotite‐type structure. The vacancy disordering and spin‐orbit correlation have not yet been introduced. Full‐potential calculations will be required because the atomic sphere approximation was adopted here. Further investigations will be carried out in future research.

In recent years, interest in spintronics has shifted from ferromagnets to antiferromagnets [52, 53]. “Altermagnets” [54, 55, 56, 57], also known as the third type of magnetic system following ferromagnets and antiferromagnets, have been the subject of much theoretical and experimental research. This new concept combines the properties of ferromagnets and antiferromagnets. Similar to antiferromagnets, the adjacent magnetic moments are aligned in opposite directions, resulting in zero magnetization throughout the material and no external magnetic field leakage. Furthermore, similar to ferromagnets, the electron energy band structure exhibits a broken time‐reversal symmetry. This is because electrons experience different “virtual magnetic fields” depending on their spin orientation, enabling the generation of a spin current with a specific spin bias.

In addition to altermagnets, FCFiMs have gained attention because the total magnetization is basically small over the entire temperature range, and especially almost zero around the magnetization compensated temperature [58]. Antiferromagnets are promising materials for applications in tunneling magnetoresistance and other devices because of their small total magnetization, which reduces the interelement interference caused by magnetic field leakage, even when devices are integrated in a small space. FCFiMs are also interesting materials for such applications, especially when they exhibit high spin polarization. Although altermagnets are characterized by spin splitting, which depends on the wavenumber, compensated ferrimagnets exhibit a uniform spin splitting in the band structure that is independent of the wavenumber. Therefore, compensated ferrimagnets are promising systems for realizing half‐metallic magnetic materials with low magnetizations. Such materials are likely to gain increased attention in spintronics research.

In this study, our research group successfully synthesized a single‐crystal sample of (Cr,Fe)_7_S_8_ with a pyrrhotite‐type structure. Experiments are currently being conducted to study the magnetocrystalline anisotropy and transport properties of this system.

## 6 Conclusion

In this study, (Cr,Fe)_46_S_54_ was fabricated at temperatures ranging from 1023 to 1423 K to optimize the sintering and quenching processing conditions for this system. In addition, single phases were obtained by sintering and quenching from 1323 to 1423 K. The XRD profiles were indexed to the pyrrhotite‐type structure. It was found that the degree of vacancy ordering differed depending on the quenching temperature, based on the assumption that the Cr and Fe atoms were randomly occupied. The specimens quenched below 1223 K exhibited the precipitation of FeCr_2_S_4_ spinel.

The single phase of the pyrrhotite shows the typical behavior of an N‐type ferrimagnet in its thermomagnetization curves, showing a fully compensated behavior. However, different magnetization compensation temperatures were observed in the 1323 and 1423 K WQ specimens. In particular, the 1323 K WQ specimen exhibited a large hysteresis in its magnetization curve obtained at 5 K, reaching a magnetic coercivity of 38 kOe. The difference in the magnetization compensated temperatures can be attributed to the slight change in the environment of the Cr and Fe atoms caused by the disordering of the vacancies in the interlayer. XPS analysis showed that the valences of the transition atoms were Cr^3+^ and Fe^2+^, which exhibited distinct roles.

First‐principles calculations suggested that a high spin polarization was maintained in the pyrrhotite and NiAs‐type structures. (Cr,Fe)S pyrrhotite is a unique magnetic material, acting as a fully compensated ferrimagnet with the potential for realizing a half‐metallic electronic state(Supporting Information).

## Conflicts of Interest

The authors declare no conflict of interest.

## Supporting information




**Supporting File**: smll74603‐sup‐0001‐SuppMat.docx.

## Data Availability

The data that support the findings of this study are available from the corresponding author upon reasonable request.

## References

[1] H. van Leuken and R. A. de Groot , “Half‐Metallic Antiferromagnets,” Physical Review Letters 74 (1995): 1171–1173, 10.1103/PhysRevLett.74.1171. 10058952

[2] W. E. Pickett , “Single Spin Superconductivity,” Physical Review Letters 77 (1996): 3185–3188, 10.1103/PhysRevLett.77.3185. 10062155

[3] J. H. Park , S. K. Kwon , and B. I. Min , “Half‐Metallic Antiferromagnetic Double Perovskites: LaAVRuO_6_ (A = Ca, Sr, and Ba),” Physical Review B 65 (2002): 174401, 10.1103/PhysRevB.65.174401.

[4] I. Galanakis , K. Özdoğan , E. Şaşıoğlu , and B. Aktaş , “Ab Initio Design of Half‐Metallic Fully Compensated Ferrimagnets: the Case of Cr_2_MnZ (Z = P, As, Sb, and Bi),” Physical Review B 75 (2007): 172405, 10.1103/PhysRevB.75.172405.

[5] E. Sasioglu , “Nonzero Macroscopic Magnetization in Half‐Metallic Antiferromagnets at Finite Temperatures,” Physical Review B 79 (2009): 100406R, 10.1103/PhysRevB.79.100406.

[6] H. Akai and M. Ogura , “Half‐Metallic Diluted Antiferromagnetic Semiconductors,” Physical Review Letters 97 (2006): 026401, 10.1103/PhysRevLett.97.026401. 16907464

[7] N. H. Long , M. Ogura , and H. Akai , “New Type of Half‐Metallic Antiferromagnet: Transition Metal Pnictides,” Journal of Physics‐Condensed Matter 21 (2009): 064241. 10.1088/0953-8984/21/6/064241 21715943

[8] R. Stinshoff , G. H. Fecher , S. Chadov , et al., “Half‐Metallic Compensated Ferrimagnetism with a Tunable Compensation Point over a Wide Temperature Range in the Mn‐Fe‐V‐Al Heusler System,” AIP Advances 7 (2017): 105009, 10.1063/1.5000351.

[9] M. Ghanathe , A. Kumar , M. D. Mukadam , and S. M. Yusuf , “Temperature Dependent Partially Compensated to Nearly Fully Compensated Magnetic State in Half‐Metallic Full Heusler Alloy, Mn_1.2_Fe_1.18_V_0.62_Al,” Journal of Magnetism and Magnetic Materials 561 (2022): 169689, 10.1016/j.jmmm.2022.169689.

[10] P. V. Midhunlal , J. A. Chelvane , D. Prabhu , R. Gopalan , and N. H. Kumar , “Mn_2_V_0.5_Co_0.5_Z (Z = Ga, Al) Mn_2_V_0.5_Co_0.5_Z (Z = Ga, Al) Heusler Alloys: High TC Compensated P‐Type Ferrimagnetism in Arc Melted Bulk and N‐type Ferrimagnetism in Melt‐Spun Ribbons,” Journal of Magnetism and Magnetic Materials 489 (2019): 165298, 10.1016/j.jmmm.2019.165298.

[11] H. Kurt , K. Rode , P. Stamenov , et al., “Cubic Mn_2_Ga Thin Films: Crossing the Spin Gap with Ruthenium,” Physical Review Letters 112 (2014): 027201, 10.1103/PhysRevLett.112.027201. 24484042

[12] L. Néel , “Propriétés Magnétiques Des Ferrites; Ferrimagnétisme et Antiferromagnétisme,” Annalen Der Physik 12 (1948): 137.

[13] S. Semboshi , R. Y. Umetsu , Y. Kawahito , and H. Akai , “A New Type of Half‐Metallic Fully Compensated Ferrimagnet,” Scientific Reports 12 (2022): 10687, 10.1038/s41598-022-14561-8. 35739287 PMC9226010

[14] H. Akai and M. Ogura , “Half‐metallic Antiferromagnets”, Japanese Patent Application No. 2008–073917, Japanese Patent Application Publication No. 2009–231471 (2008).

[15] F.‐C. Hsu , J.‐Y. Luo , K.‐W. Yeh , et al., “Superconductivity in the PbO‐Type Structure α‐FeSe,” PNAS 105 (2008): 14262. 18776050 10.1073/pnas.0807325105 PMC2531064

[16] E. F. Bertaut , “Contribution à L'étude Des Structures Lacunaires: La Pyrrhotine,” Acta Crystallographica 6 (1953): 557–561, 10.1107/S0365110X53001502.

[17] M. Tokonami , K. Nishiguchi , and N. Morimoto , “Crystal Structure of a Monoclinic Pyrrhotite (Fe_7_S_8_),” Automated Mineralogy 57 (1972): 1066.

[18] M. Venkatraman , “Chromium‐Sulfur Binary Phase Diagram,” in ASM Alloy Phase Diagram Database, (Eds. P. Villars , H. Okamoto , and K. Cenzual ), Sec. Eds. Materials Park, OH, USA: ASM International, 1990.

[19] P. Waldner , “Iron‐Sulfur Binary Phase Diagram,” in ASM Alloy Phase Diagram Database, (Eds. P. Villars , H. Okamoto , and K. Cenzual ), Sec. Eds. Materials Park, OH, USA: ASM International, 2005.

[20] V. Raghavan , “Chromium‐Iron‐Sulfur Ternary, Isothermal Section,” in ASM Alloy Phase Diagram Database, (Eds. P. Villars , H. Okamoto , and K. Cenzual ), Sec. Eds. Materials Park, OH, USA: ASM International, 1988.

[21] A. P. Ramirez , R. J. Cava , and J. Krajewski , “Colossal Magnetoresistance in Cr‐Based Chalcogenide Spinels,” Nature 386 (1997): 156–159, 10.1038/386156a0.

[22] L. F. Feiner , “Unified Description of the Cooperative Jahn‐Teller Effect in FeCr_2_S_4_ and the Impurity Jahn‐Teller Effect in CoCr_2_S_4_:Fe^2+^ ,” Journal of Physics C: Solid State Physics 15 (1982): 1515–1524, 10.1088/0022-3719/15/7/017.

[23] M. Eibschutz , S. Shtrikman , and Y. Tenenbaum , “Magnetically Induced Electric Field Gradient in Tetrahedral Divalent Iron: FeCr_2_S_4_ ,” Physics Letters A 24 (1967): 563.

[24] M. R. Spender and A. H. Morrish , “A Low Temperature Transition in FeCr_2_S_4_ ,” Solid State Communications 11 (1972): 1417–1421, 10.1016/0038-1098(72)90556-X.

[25] L. Brossard , J. L. Dormann , L. Goldstein , P. Gibart , and P. Renaudin , “Second‐Order Phase Transition in FeCr_2_S_4_ Investigated by Mössbauer Spectroscopy: an Example of Orbital Para‐to‐Ferromagnetism Transition,” Physical Review B 20 (1979): 2933–2944, 10.1103/PhysRevB.20.2933.

[26] A. M. van Diepen and R. P. van Stapele , “Ordered Local Distortions in Cubic FeCr_2_S_4_ ,” Solid State Communications 13 (1973): 1651–1653, 10.1016/0038-1098(73)90258-5.

[27] K. Ohgushi , S. Miyasaka , and Y. Tokura , “Anisotropic Anomalous Hall Effect of Topological Origin in Carrier‐Doped FeCr_2_S_4_ ,” Journal of the Physical Society of Japan 75 (2006): 013710, 10.1143/JPSJ.75.013710.

[28] J. Bertinshaw , C. Ulrich , A. Günther , et al., “FeCr_2_S_4_ in Magnetic Fields: Possible Evidence for a Multiferroic Ground State,” Scientific Reports 4 (2014): 6079, 10.1038/srep06079. 25123960 PMC4133713

[29] L. Lin , H. X. Zhu , X. M. Jiang , et al., “Coupled Ferroelectric Polarization and Magnetization in Spinel FeCr_2_S_4_ ,” Scientific Reports 4 (2014): 6530. 25284432 10.1038/srep06530 PMC4185382

[30] V. Felea , S. Yasin , A. Günther , et al., “Ultrasound Study of FeCr_2_S_4_ in High Magnetic Fields,” Journal of Physics‐Condensed Matter 48 (2014): 486001. 10.1088/0953-8984/26/48/486001 25366066

[31] J. Deisenhofer , F. Mayr , M. Schmidt , A. Loidl , and V. Tsurkan , “Infrared‐Active Phonons in the Ferrimagnetic and Multiferroic Phases of FeCr_2_S_4_: Evidence for Structural Distortions,” Physical Review B 100 (2019): 144428, 10.1103/PhysRevB.100.144428.

[32] L. Prodan , S. Yasin , A. Jesche , et al., “Unusual Field‐Induced Spin Reorientation in FeCr_2_S_4_: Field Tuning of the Jahn‐Teller State,” Physical Review B 104 (2021): L020410, 10.1103/PhysRevB.104.L020410.

[33] M. Ogura and H. Akai , “The Full Potential Korringa–Kohn–Rostoker Method and Its Application in Electric Field Gradient Calculations,” Journal of Physics: Condensed Matter 17 (2006): 5741–5755, 10.1088/0953-8984/17/37/011. 32397046

[34] J. P. Perdew , K. Burke , and M. Ernzerhof , “Generalized Gradient Approximation Made Simple,” Physical Review Letters 77 (1996): 3865–3868, 10.1103/PhysRevLett.77.3865. 10062328

[35] H. Akai , “Electronic Structure Ni–Pd Alloys Calculated by the Self‐Consistent KKR‐CPA Method,” Journal of the Physical Society of Japan 51 (1982): 468–474, 10.1143/JPSJ.51.468.

[36] H. Akai , “Fast Korringa‐Kohn‐Rostoker Coherent Potential Approximation and Its Application to FCC Ni‐Fe Systems,” Journal of Physics: Condensed Matter 1 (1989): 8045–8064, 10.1088/0953-8984/1/43/006.

[37] V. V. Sokolovich and O. A. Bayukov , “Mössbauer Spectra of Fe_x_Cr_1 − x_S solid Solutions,” Physics of the Solid State 49 (2007): 1920–1922, 10.1134/S1063783407100174.

[38] T. Kanomata , K. Shirakawa , and T. Kaneko , “Effect of Hydrostatic Pressure on the Curie Temperature of FeCr_2_S_4_ and CoCr_2_S_4_ ,” Journal of the Physical Society of Japan 54 (1985): 334–338, 10.1143/JPSJ.54.334.

[39] V. Tsurkan , J. Hemberger , M. Klemm , et al., “Ac Susceptibility Studies of Ferrimagnetic FeCr_2_S_4_ Single Crystals,” Journal of Applied Physics 90 (2001): 4639–4644, 10.1063/1.1405827.

[40] C. Broquetas Colominas , R. Ballestracci , and G. Roult , “Étude Par Diffraction Neutronique Du Spinelle FeCr_2_S_4_ ,” Journal de Physique 25 (1964): 526–528, 10.1051/jphys:01964002505052600.

[41] G. Shirane , D. E. Cox , and S. J. Pickart , “Magnetic Structures in FeCr_2_S_4_ and FeCr_2_O_4_ ,” Journal of Applied Physics 35 (1964): 954.

[42] T.‐H. Wu , H. Fu , R. A. Hajjar , T. Suzuki , and M. Mansuripur , “Measurement of Magnetic Anisotropy Constant for Magneto‐Optical Recording Media: a Comparison of Several Techniques,” Journal of Applied Physics 73 (1993): 1368–1376, 10.1063/1.353256.

[43] J. Ostorero , M. Escorne , A. Pecheron‐Guegan , F. Soulette , and H. L. Gall , “Dy_3_Fe_5_O_12_ Garnet Thin Films Grown from Sputtering of Metallic Targets,” Journal of Applied Physics 75 (1994): 6103–6105, 10.1063/1.355474.

[44] D. J. Webb , A. F. Marshall , Z. Sun , T. H. Geballe , and R. M. White , “Coercivity of a Macroscopic Ferrimagnet near a Compensation Point,” IEEE Transactions on Magnetics 24 (1988): 588–592, 10.1109/20.43988.

[45] S. Demirtas , M. R. Hossu , R. E. Camley , H. C. Mireles , and A. R. Koymen , “Tunable Magnetic Thermal Hysteresis in Transition Metal (Fe, Co, CoNi)/Rare Earth (Gd) Multilayers,” Physical Review B 72 (2005): 184433.

[46] L. T. Tsymbal , Y. B. Bazaliy , V. N. Derkachenko , et al., “Magnetic and Structural Properties of Spin‐Reorientation Transitions in Orthoferrites,” Journal of Applied Physics 101 (2007): 123919, 10.1063/1.2749404.

[47] E. E. Zubov , “Crystal Field and Origin of Exchange Bias in Compensated Rare‐Earth Ferrimagnets,” Journal of Magnetism and Magnetic Materials 551 (2022): 169153, 10.1016/j.jmmm.2022.169153.

[48] M. C. Biesinger , C. Brown , J. R. Mycroft , R. D. Davidson , and N. S. McIntyre , “X‐Ray Photoelectron Spectroscopy Studies of Chromium Compounds,” Surface and Interface Analysis 36 (2004): 1550–1563, 10.1002/sia.1983.

[49] M. C. Biesinger , B. P. Payne , A. P. Grosvenor , L. W. M. Lau , A. R. Gerson , and R. St C Smart , “Resolving Surface Chemical States in XPS Analysis of First Row Transition Metals, Oxides and Hydroxides: Cr, Mn, Fe, Co and Ni,” Applied Surface Science 257 (2011): 2717–2730, 10.1016/j.apsusc.2010.10.051.

[50] M. V. Morales‐Gallardo , A. M. Ayala , M. Pal , M. A. Cortes Jacome , J. A. Toledo Antonio , and N. R. Mathews , “Synthesis of Pyrite FeS_2_ Nanorods by Simple Hydrothermal Method and Its Photocatalytic Activity,” Chemical Physics Letters 660 (2016): 93–98, 10.1016/j.cplett.2016.07.046.

[51] A. V. Powell , P. Vaqueiro , K. S. Knight , L. C. Chapon , and R. D. Sánchez , “Structure and Magnetism in Synthetic Pyrrhotite Fe_7_S_8_: a Powder Neutron‐Diffraction Study,” Physical Review B 70 (2004): 014415, 10.1103/PhysRevB.70.014415.

[52] J. Želenznŷ , P. Wadley , K. Olejník , A. Hoffmann , and H. Ohno , “Spin Transport and Spin Torque in Antiferromagnetic Devices,” Nature Phys 14 (2018): 220.

[53] D. Xiong , Y. Jiang , K. Shi , et al., “Antiferromagnetic Spintronics: an Overview and Outlook,” Fundamental Research 2 (2022): 522–534, 10.1016/j.fmre.2022.03.016. 38934004 PMC11197578

[54] L. Šmejkal , J. Sinova , and T. Jungwirth , “Emerging Research Landscape of Altermagnetism,” Physical Review X 12 (2022): 040501.

[55] L. Šmejkal , J. Sinova , and T. Jungwirth , “Beyond Conventional Ferromagnetism and Antiferromagnetism: a Phase with Nonrelativistic Spin and Crystal Rotation Symmetry,” Physical Review X 12 (2022): 031042.

[56] Y. Liu , S.‐D. Guo , Y. Li , and C.‐C. Liu , “Two‐Dimensional Fully Compensated Ferrimagnetism,” Physical Review Letters 134 (2025): 116703, 10.1103/PhysRevLett.134.116703. 40192380

[57] N. Cheng , H. Cheng , X. Zhao , G. Hu , X. Yuan , and J. Ren , “Ferroelectric Polarization Manipulates the Layer‐Polarized Anomalous Hall Effect in Bilayers with Fully Compensated Ferrimagnetism,” Physical Review B 111 (2025): 195154, 10.1103/PhysRevB.111.195154.

[58] T. Kawamura , K. Yoshimi , K. Hashimoto , A. Kobayashi , and T. Misawa , “Compensated Ferrimagnets with Colossal Spin Splitting in Organic Compounds,” Physical Review Letters 132 (2025): 156502, 10.1103/PhysRevLett.132.156502. 38682965

