# Supplementary material for: Fully Compensated Ferrimagnetic Properties of (Cr,Fe)S Compound with a Pyrrhotite‐Type Structure
Source: Small. 2026 Aug 3;22(51):e74603. doi: 10.1002/smll.74603 (PMC13568839; doi:10.1002/smll.74603)
Supplement: Supplementary file 1 [file SMLL-22-e74603-s001.docx]

Supplemental Information

*Model-I*

XRD patterns were further refined by the Rietveld method with Z-Code software [S1,S2]. Two patterns of the models were considered for the specimens obtained by quenching at 1323 and 1423 K for Cr_23_Fe_23_S_54_ compound (1323 K WQ and 1423 K WQ, respectively). In the first model for 1323 K WQ, the vacancy is assumed to be distributed in site 4 and site 4*. Fitting results became better when the occupation at the site 2 is not complete 1 rather 0.9. To fix the composition as (Cr,Fe)_7_S_8_, the occupancies at the 4 and site 4* were set to 0.6. Under the assumption, the positions of all atoms were refined. The resultant parameters were listed in Suppl. Table 1 and the fitted patterns were shown in Suppl. Fig. 1. The result is just the one kind of the possibility for the structure model. Further refinement will be required such as neutron diffraction, because Cr and Fe atoms were assumed here to be randomly distributed at the sites 1-4. The lattice parameters were *a* = 1.1940, *b* = 0.6887, *c* = 1.2898 nm and *β* = 117.5, the goodness-of-fit indicator *S* = 1.4310.

Suppl. Table 1

List of the occupied atoms at each site, the Wyckoff position, atomic position (*x*, *y*, *z*) and the occupancy obtained by the Rietveld analysis based on the Pyrrhotite-type structure for the specimen 1323 K WQ.

| Site | Atoms | Wyckoff | x | y | z | Occ. |
| --- | --- | --- | --- | --- | --- | --- |
| Site 1 | Cr0.5 + Fe0.5 | 8f | 0.1337 | 0.1220 | 0.9969 | 1 |
| Site 2 | Cr0.5 + Fe0.5 | 8f | 0.2525 | 0.1213 | 0.2488 | **0.9** |
| Site 3 | Cr0.5 + Fe0.5 | 8f | 0.3678 | 0.1222 | 0.5037 | 1 |
| Site 4 | Cr0.5 + Fe0.5 | 4e | 0 | 0.3973 | 0.25 | **0.6** |
| Site 4* | Cr0.5 + Fe0.5 | 4e | 0 | 0.9025 | 0.25 | **0.6** |
| Site 5 | S | 8f | 0.8989 | 0.1250 | 0.8781 | 1 |
| Site 6 | S | 8f | 0.3537 | 0.1252 | 0.1226 | 1 |
| Site 7 | S | 8f | 0.8519 | 0.1284 | 0.1220 | 1 |
| Site 8 | S | 8f | 0.5994 | 0.1285 | 0.6227 | 1 |

Suppl. Fig. 1 XRD pattern and the Rietveld analysis for the specimen 1323 K WQ.

*Model-II*

This model was used for the refinement for the XRD pattern in the specimen 1423 K WQ. Here, the vacancies were assumed to be distributed at the sites 2, 4 and 4*, therefore, the sits occupancies were set to 0.75. This assumption was the best in the present analyses. Because these sites locate in the same layer, it is natural the disordering may be occurred between them. As in the same manner with the model-I, the atomic positions were refined after fix the site occupancies. The lattice parameters were *a* = 1.1968, *b* = 0.6888, *c* = 1.2938 nm and *β* = 117.5, the goodness-of-fit indicator *S* = 1.2902.

Suppl. Table 2

List of the occupied atoms at each site, the Wyckoff position, stomic position (*x*, *y*, *z*) and the occupancy obtained by the Rietveld analysis based on the Pyrrhotite-type structure for the specimen 1423 K WQ.

|  |  | Wyckoff | x | y | z | Occ. |
| --- | --- | --- | --- | --- | --- | --- |
| Site 1 | Cr0.5 + Fe0.5 | 8f | 0.1271 | 0.1170 | 0.9963 | 1 |
| Site 2 | Cr0.5 + Fe0.5 | 8f | 0.2503 | 0.1155 | 0.2496 | **0.75** |
| Site 3 | Cr0.5 + Fe0.5 | 8f | 0.3614 | 0.1172 | 0.5062 | 1 |
| Site 4 | Cr0.5 + Fe0.5 | 4e | 0 | 0.3911 | 0.25 | **0.75** |
| Site 4* | Cr0.5 + Fe0.5 | 4e | 0 | 0.8809 | 0.25 | **0.75** |
| Site 5 | S | 8f | 0.8993 | 0.1277 | 0.8781 | 1 |
| Site 6 | S | 8f | 0.3561 | 0.1332 | 0.1233 | 1 |
| Site 7 | S | 8f | 0.8613 | 0.1325 | 0.1254 | 1 |
| Site 8 | S | 8f | 0.600 | 0.1288 | 0.6206 | 1 |

Suppl. Fig. 2 XRD pattern and the Rietveld analysis for the specimen 1423 K WQ.

[S1] R. Oishi, M. Yonemura, Y. Nishimaki, S. Torii, A. Hoshikawa, T. Ishigaki, T. Morishima, K. Mori and T. Kamiyama, “Rietveld analysis software for J-PARC”, Nuclear Instruments and Methods A600, 94–96 (2009)

[S2] R. Oishi-Tomiyasu, M. Yonemura, T. Morishima, A. Hoshikawa, S. Torii, T. Ishigaki, T. Kamiyama, “Application of matrix decomposition algorithms for singular matrices to the Pawley method in Z-Rietveld”, J. Appl. Cryst. 45, 299–308 (2012)
